# Ethanol Inhibits Activation of NLRP3 and AIM2 Inflammasomes in Human Macrophages–A Novel Anti-Inflammatory Action of Alcohol

**DOI:** 10.1371/journal.pone.0078537

**Published:** 2013-11-11

**Authors:** Katariina Nurmi, Juhani Virkanen, Kristiina Rajamäki, Katri Niemi, Petri T. Kovanen, Kari K. Eklund

**Affiliations:** 1 Wihuri Research Institute, Helsinki, Finland; 2 University of Helsinki, Department of Geosciences and Geography, Helsinki, Finland; 3 Division of Rheumatology, Department of Medicine, Helsinki University Central Hospital, Helsinki, Finland; University of Milan, Italy

## Abstract

**Objective:**

In the pathogenesis of coronary atherosclerosis, local macrophage-driven inflammation and secretion of proinflammatory cytokines, interleukin-1β (IL-1β) in particular, are recognized as key factors. Moderate alcohol consumption is associated with a reduced risk of coronary artery disease mortality. Here we examined in cultured human macrophages whether ethanol modulates the intracellular processes involved in the secretion of IL-1β.

**Results:**

Ethanol decreased dose-dependently the production of mature IL-1β induced by activators of the NLRP3 inflammasome, i.e. ATP, cholesterol crystals, serum amyloid A and nigericin. Ethanol had no significant effect on the expression of *NLRP3* or *IL1B* mRNA in LPS-primed macrophages. Moreover, secretion of IL-1β was decreased in parallel with reduction of caspase-1 activation, demonstrating that ethanol inhibits inflammasome activation instead of synthesis of pro-IL-1β. Acetaldehyde, a highly reactive metabolite of ethanol, had no effect on the ATP-induced IL-1β secretion. Ethanol also attenuated the secretion of IL-1β triggered by synthetic double-stranded DNA, an activator of the AIM2 inflammasome. Ethanol conferred the inhibitory functions by attenuating the disruption of lysosomal integrity and ensuing leakage of the lysosomal protease cathepsin B and by reducing oligomerization of ASC.

**Conclusion:**

Ethanol-induced inhibition of the NLRP3 inflammasome activation in macrophages may represent a biological pathway underlying the protective effect of moderate alcohol consumption on coronary heart disease.

## Introduction

Light to moderate consumption of alcoholic beverages is associated with a reduced risk of coronary artery disease (CAD) [1]. This protective effect has been largely attributed to the ethanol-induced increase of circulating HDL-cholesterol levels and to the antioxidant properties of polyphenols found in high amounts particularly in red wine [2], [3], [4]. However, the reduced CAD risk associates also with the consumption of other kinds of alcoholic beverages, and, moreover, with the slow-oxidizing allele of alcohol dehydrogenase, so strongly suggesting that ethanol itself is largely responsible for the observed cardioprotective effect [5], [6], [7].

Increased level of the high-sensitivity C-reactive protein (hs-CRP), indicator of a chronic low-degree inflammation in the body, associates with increased CAD risk [8]. Compared to abstainers, the levels of hs-CRP tend to be lower among moderate alcohol consumers [9]. Since atherosclerosis is an inflammatory disease, a local anti-inflammatory action of moderate alcohol consumption in the diseased coronary arteries could contribute to the observed reductions in hs-CRP, and in morbidity and mortality from CAD [10], [11]. In atherosclerotic lesions, macrophages represent the major source of proinflammatory mediators, such as interleukin-1β (IL-1β) [12]. IL-1β is a key cytokine in the atherosclerotic inflammation of human coronary arteries, and the levels of IL-1β correlate positively with the severity of CAD [13]. Supporting evidence for a role of IL-1β in atherogenesis has been obtained from experiments in atherosclerosis-prone mice. Thus, in ApoE−/− mice deficiency of IL-1β decreased the extent of atherosclerosis [14]. Furthermore, the overexpression of IL-1 receptor antagonist in ApoE −/− mice decreased the atherosclerotic lesion size [15].

The production of IL-1β is tightly regulated. Thus, two separate signals are required for the secretion of the active mature cytokine. The first signal is produced by the activation of a pattern recognition receptor, such as Toll-Like Receptor 4 (TLR4), which induces the production of pro-IL-1β, and the second signal activates caspase-1, an enzyme that proteolytically cleaves pro-IL-1β into its mature form, which is then secreted from the macrophages [16]. The activation of caspase-1 is mediated by intracellular multiprotein complexes, the inflammasomes. Several different inflammasomes have been described, of which the NLRP3 (nucleotide-binding domain and leucine-rich repeat containing family, pyrin domain containing 3) inflammasome is the most extensively studied. The NLRP3 receptor is activated by diverse substances, including pore-forming toxins, extracellular ATP [17], microbial DNA and RNA [18], [19], inhaled particulates [20], uric acid, and notably, also cholesterol crystals [21], [22], [23]. The activated NLRP3 receptor oligomerizes and recruits caspase-1 through the adaptor protein ASC (apoptosis-associated speck-like protein containing a caspase-recruitment domain), thus forming an active NLRP3 inflammasome complex [24], [25]. Potassium efflux, leakage of cathepsin B from lysosomes, as well as the generation of reactive oxygen species (ROS), all have been implicated as downstream effectors leading to the activation of the NLRP3 inflammasome [26].

The significance of the inflammasomes in the regulation of inflammatory reactions in the human body is exemplified by autoinflammatory diseases, many of which are caused by excessive activation of the NLRP3 inflammasome [27]. Activation of the NLRP3 inflammasome with ensuing increased production of IL-1β has been assigned a key role also in the pathogenesis of several common chronic diseases with inflammatory features. These include gout, and more recently also atherosclerosis [21], [22], [23], type II diabetes, and obesity [28], [29]. The above pieces of information led us to postulate that the anti-atherogenic effects of ethanol might be, at least partly, due to the inhibition of the innate immune responses of macrophages to the inflammasome-activating stimuli. Consistent with this hypothesis, we demonstrate here that ethanol significantly inhibits the secretion of IL-1β from cultured human macrophages stimulated by diverse NLRP3 inflammasome activators.

## Materials and Methods

### Materials

Macrophage-SFM and fetal bovine serum were from Gibco, recombinant mononuclear phagocyte colony-stimulating factor (M-CSF) and recombinant granulocyte macrophage colony-stimulating factor (GM-CSF) were from Biosite. RPMI 1640, L-glutamine, HEPES buffer, and penicillin-streptomycin antibiotics were from Lonza. UltraPure lipopolysaccharides (tlrl-pelps) were purchased from InvivoGen and lipopolysaccharides (LPS) from Sigma. Polyclonal antibodies used in Western blotting against human caspase-1 p10, human caspase-8 and human IL-1β were from Santa Cruz Biotechnology, monoclonal anti-human beta-actin was from Abcam and anti-human ASC from MBL international. HRP conjugates used as secondary antibodies in Western blotting; goat-anti-rabbit IgG (H+L) was from Zymed Laboratories, goat-anti-mouse from Dako and biotinylated goat-anti-rabbit from Zymed laboratories, Alexa fluor 488 was used as a secondary antibody for immunocytochemistry (Invitrogen). *N*-acetyl-L-cysteine (NAC), phorbol 12-myristate 13-acetate (PMA), nigericin sodium salt, 4-methylpyrazole and poly(deoxyadenylic-thymidylic) acid sodium salt [Poly (dA:dT)] were all from Sigma. Recombinant human apo-serum amyloid A (SAA, isotype 1α), containing <0.1 ng endotoxin per mg protein, was from Peprotech. Cholesterol for preparation of cholesterol crystals (CHC) was from Sigma. Sterile 99% pure ethanol (ALTIA) and anhydrous acetaldehyde (Fluka, Sigma) were used in all experiments.

### Crystal Preparation

Cholesterol was crystallized as previously described by Rajamäki et al. [22]. Crystal preparations were tested to be below detection limits (<0.03 endotoxin units/ml) by Pyrogent Gel Clot LAL Assay (Lonza). Crystals were stored at −20°C.

### Cell Cultures

#### Primary human macrophages

Human white blood cell preparations (buffy coats) from healthy donors, were purchased from the Finnish Red Cross Blood Service (Helsinki, Finland). Monocytes were isolated and differentiated into macrophages using GM-CSF (10 ng/ml) or (M-CSF, 50 ng/ml) as previously described by Nakanishi et al. [30].

#### THP-1 cells

Human monocytic leukemia cell line (THP-1) was purchased from the American Type Culture Collection (Manassas, VA; cat. TIB-202). Cells were cultured in RPMI 1640 supplemented with 2 mM L-glutamine, 10% fetal bovine serum, 25 mM HEPES, 100 U/ml penicillin, and 100 µg/ml streptomycin. To induce monocyte to macrophage differentiation, the THP-1 cells were cultured for 72 h in the presence of 100 nM PMA.

### Ethics Statement

Buffy coats were obtained from healthy blood donors, who had signed an informed consent document. The buffy coats were by-products from the preparation of blood products for clinical use. The use of buffy coats in monocyte isolation was approved by the Finnish Red Cross Blood Service.

### Measurement of Cell Death

Trypan blue stain (0.2% w/v Trypan blue in PBS, 2 min) was used to evaluate cell death after experiments. Cytotoxicity Detection Kit based on lactate dehydrogenase release (Roche) was used according to manufactureŕs protocols.

### Activation of Inflammasomes in Cultured Macrophages

Briefly, to activate the NLRP3 inflammasome human primary macrophages or THP-1 cells were first primed with the TLR4 agonist LPS (1 µg/ml, 3 h) in the indicated experiments. After priming, the cells were washed twice and preincubated for indicated times in the presence of varying concentrations of ethanol (2.5‰, 5‰, 10‰, and 20‰ corresponding to 43 mM, 86 mM, 171 mM, and 343 mM, respectively). Indicated concentrations of ethanol were present during the activation of the inflammasomes, unless otherwise stated.

#### Activation of the NLRP3 inflammasome

Human primary macrophages were preincubated at the indicated concentrations of ethanol for 1 h, and then stimulated with cholesterol crystals (1 mg/ml, 20 h) [22]. After an 1-h preincubation in the presence of the indicated concentrations of ethanol, THP-1 cells were activated for 5 h and primary macrophages for 20 h with SAA (3 µg/ml), while SAA-induced activation of the NLRP3 inflammasome does not require a separate priming signal [31]. A 3-h preincubation at the indicated concentrations of ethanol preceded the activations with ATP (5 mM, 30 min) and nigericin (4 µM, 1 h). To inhibit ROS formation, 0.5 µM NAC was added to the cell culture medium 1 h before the ethanol incubation. To inhibit alcohol dehydrogenase, 4-methylpyrazole (1 µM) was added to cell culture medium 2 h before ethanol incubation.

#### Activation of the AIM2 inflammasome

THP-1 cells were incubated in the presence of the indicated concentrations of ethanol for 30 min prior to the activation of the AIM2 inflammasome with synthetic dsDNA (0.1 µg/ml, 6 h). Poly (dA:dT) was transfected with Lipofectamine™ LTX and Plus Reagent (Invitrogen) according to the manufacturer’s instructions.

### Enzyme-linked Immunosorbent Assay

The concentrations of the secreted IL-1β and IL-18 in the cell culture supernatants were assessed using a commercial ELISA assay according to the manufacturer’s instructions. The IL-18 assay was purchased from Medical & Biological Laboratories, and IL-1β was from R&D Systems.

### Western Blotting

Cell culture media were concentrated using Vivaspin 6 centrifugal concentrator with a 5 kDa cut-off (Sartorius), after which immunoblot analysis was performed. Adherent cells were lysed using ice-cold Radio-Immuneprecipitation Assay Buffer (RIPA) (50 mM Tris - 150 mM NaCl - 2 mM EDTA - 1% NP-40 - 0.1% SDS) supplemented with 1× Protease Inhibitor Cocktail (Roche). The lysates were incubated on ice for 20 min, subjected to a 15 s water bath sonication and centrifuged. Concentrated media samples and cell lysates (30–60 µg of total protein) were subjected to SDS-PAGE and transferred onto Hybond-C Extra nitrocellulose membrane (Amersham). IL-1β and caspase-1 Western blot membranes were incubated o/n with the primary antibodies (diluted in 5% non-fat milk – 0.1% Tween-20 – TBS) at +4°C. ASC Western blot membranes were incubated o/n with the primary antibodies (diluted in 1% non-fat milk – PBS) at +4°C. Caspase-8 and beta-actin Western blot membranes were incubated o/n with the primary antibodies (diluted in 5% bovine serum albumin –0.05% Tween-20 – TBS). Signal was detected with the Super Signal West Pico chemiluminescent substrate (Thermo Fisher Scientific). In detection of caspase-8 and beta-actin Western blots Clarity Western ECL substrate (BIO-RAD) was used.

### Quantitative Real-time RT-PCR

Total cellular RNA was isolated and purified using RNeasy mini kit (Qiagen). Thereafter 0.9 µg of RNA was reverse-transcribed into cDNA using random hexamers dNTP and MMLV reverse transcriptase (Promega). To analyze mRNA expression, quantitative real-time RT-PCR was performed in duplicates using TaqMan Universal PCR Master Mix (Applied Biosystems) for *IL1B, NLRP3, AIM2,* and *GAPDH*, with gene-specific primers and probes on ABI PRISM 7500 sequence detector system (Applied Biosystems). The data were developed with Sequence Detector System software (version 1.4, Applied Biosystems) and the threshold values (Cts) were selected according to the manufacturer’s guidelines. For data normalization, an endogenous control defining cDNA input (*GAPDH*) was used, and the relative units for gene expression were calculated by the comparative Ct method [32]. The gene-specific primers and probes are listed in Table S1.

### Measurement of Cholesterol Crystal Uptake

Human primary macrophages were preincubated in the presence of the indicated concentrations of ethanol prior to the addition of cholesterol crystals (1 mg/ml, 20 h). Cellular lipids were extracted from primary macrophages with hexane-isopropanol (3∶2, v/v). The solvent was evaporated under nitrogen and the lipids were redissolved in chloroform-methanol (2∶1, v/v). Samples were applied onto silica-coated thin layer chromatography (TLC) plates, hexane/diethyl ether/concentrated acetic acid/H_2_O (130∶30:2∶0.5, v/v) was used as the mobile phase. The lipids were visualized by dipping the TLC plate into CuSO4 (3%)/H_2_PO_4_ (8%) and subsequently heating the plate at 180°C. The bands were scanned with TLC Scanner3 (CAMAG) and analyzed using TLC Evaluation Software (CAMAG).

### Cathepsin B Imaging and Activity Measurement

#### Cathepsin B and lysosome imaging

To visualize the cholesterol crystal-induced release of cathepsin B from disrupted lysosomes [22], THP-1 cells were cultured on coverslips. The cells were preincubated in the presence of 10‰ (171 mM) ethanol for 1 h before the activation with cholesterol crystals (1 mg/ml, 6 h) and then stained with a fluorescent cell-permeable selective cathepsin B substrate z-Arg-Arg-cresyl violet and Hoechst stain (0.75% v/v) or acridine orange (5 µM, 0.5% v/v) according to manufacturer’s protocols (AK-125, BIOMOL). Live cells were examined with an epifluorescence microscope.

#### Cathepsin B/L activity measurement

To assess the nigericin-induced release of cathepsin B from intact lysosomes [33], LPS-primed (1 µg/ml, 3 h) THP-1 cells were preincubated in the presence of indicated concentrations of ethanol for 3 h before the activation of the NLRP3 inflammasome with nigericin (4 µM, 1 h). Activity measurement was conducted as previously described by Luheshi et al. [34], except that activity was measured from incubation medium. Briefly, after incubation the media were collected and mixed (1∶1) with the 2× reaction buffer (0.2 M sodium acetate buffer, 4 mM EDTA, 4 mM DTT added just before use, pH 5.5) Cathepsin B/L-dependent hydrolysis of the fluorogenic substrate Z-Phe-Arg-AMC (Enzo Life Sciences) (40 µM) was measured following the increase in fluorescence (excitation 355 nm, emission 486 nm) at four different time points after incubation at 37°C.

### ASC Imaging

THP-1 cells were cultured on 8-well chamber slides (Nunc). The cells were preincubated in the presence of 10‰ ethanol (171 mM, 3 h) before activating the NLRP3 inflammasome with nigericin (4 µM, 1 h). After the activation the cells were fixed with 4% PFA in PBS for 20 min, blocked and permeabilized with 3% normal goat serum and 0.1% saponin for 45 min. Primary ASC antibody was incubated o/n at +4°C. Secondary antibody, Alexa fluor 488, was incubated for 1 h at RT, and the nuclei were stained with DAPI. Cells were mounted with Fluorescence mounting medium (Dako). The slides were visualized using an epifluorescence microscope. To quantify the extent of speck formation, the number of specks per field was counted and divided by the number of cells per field, and expressed as percentage. Cells from 10 different fields (on average 250 cells/field) were counted for each experiment (n = 4).

### ICP-MS Measurement

THP-1 cells were preincubated in the presence of 10‰ ethanol (171 mM, 3 h) prior to the activation of the NLRP3 inflammasome with nigericin (4 µM, 1 h). The cells were lysed in 70% nitric acid (Romil-SpA) at RT o/n. Analyses were performed in the Department of Geosciences and Geography at the University of Helsinki using the Agilent 7500 ce/cx ICP-MS. Samples were diluted to 5% HNO_3_ and analyzed according to ISO 17294-2 [35] in helium mode. External K calibration was performed between 0 and 24 ppm. Sc and Ge were used as internal standards. Data for each sample were acquired from 6 parallel wells and averaged. National Institute of Standards and Technology standard reference material (NIST-1577C) was used to measure elemental recovery of >90%. Elemental recovery in the measurements was 101%.

### Statistical Analysis

**Figure 1:**
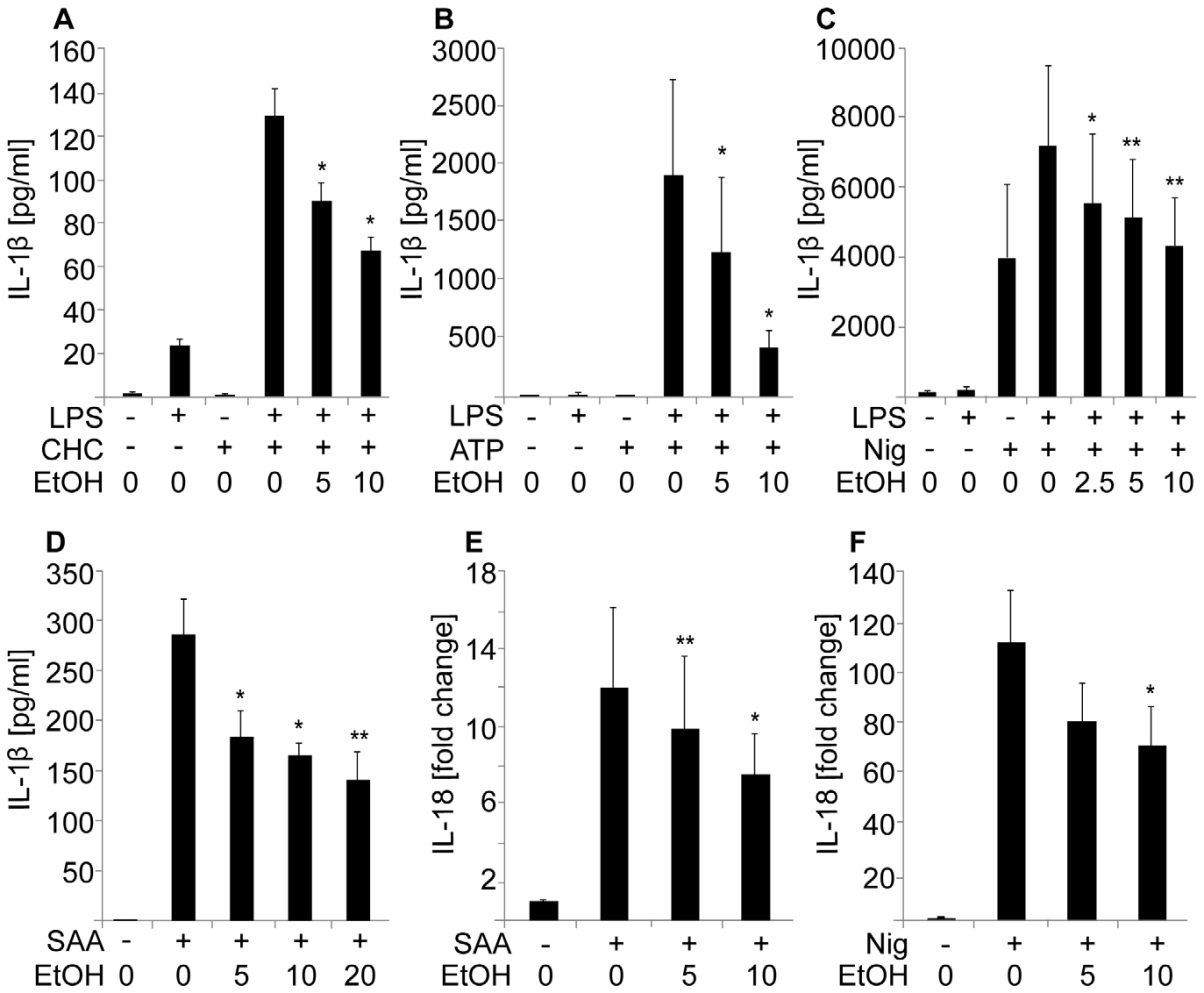
Ethanol inhibits dose-dependently the secretion of IL-1β and IL-18. LPS-primed human macrophages were preincubated in the presence of the indicated concentrations of ethanol (EtOH: 2.5‰, 5‰, 10‰, and 20‰ corresponding to 43 mM, 86 mM, 171 mM, and 343 mM, respectively) prior to the activation of the NLRP3 inflammasome with (**A**) cholesterol crystals (CHC), (**B**) ATP or (**C**) nigericin (Nig). For activation with (**D**) SAA no LPS priming was used. To measure the secretion of IL-18, THP-1 cells were preincubated in the presence of the indicated concentrations of ethanol and activated with (**E**) SAA or (**F**) nigericin. Cytokine secretion by human primary macrophages (A,B,D) and THP-1 cells (C,E,F) was analyzed by ELISA. The results are expressed as means ± s.e.m from at least 4 individual experiments.

**Figure 2:**
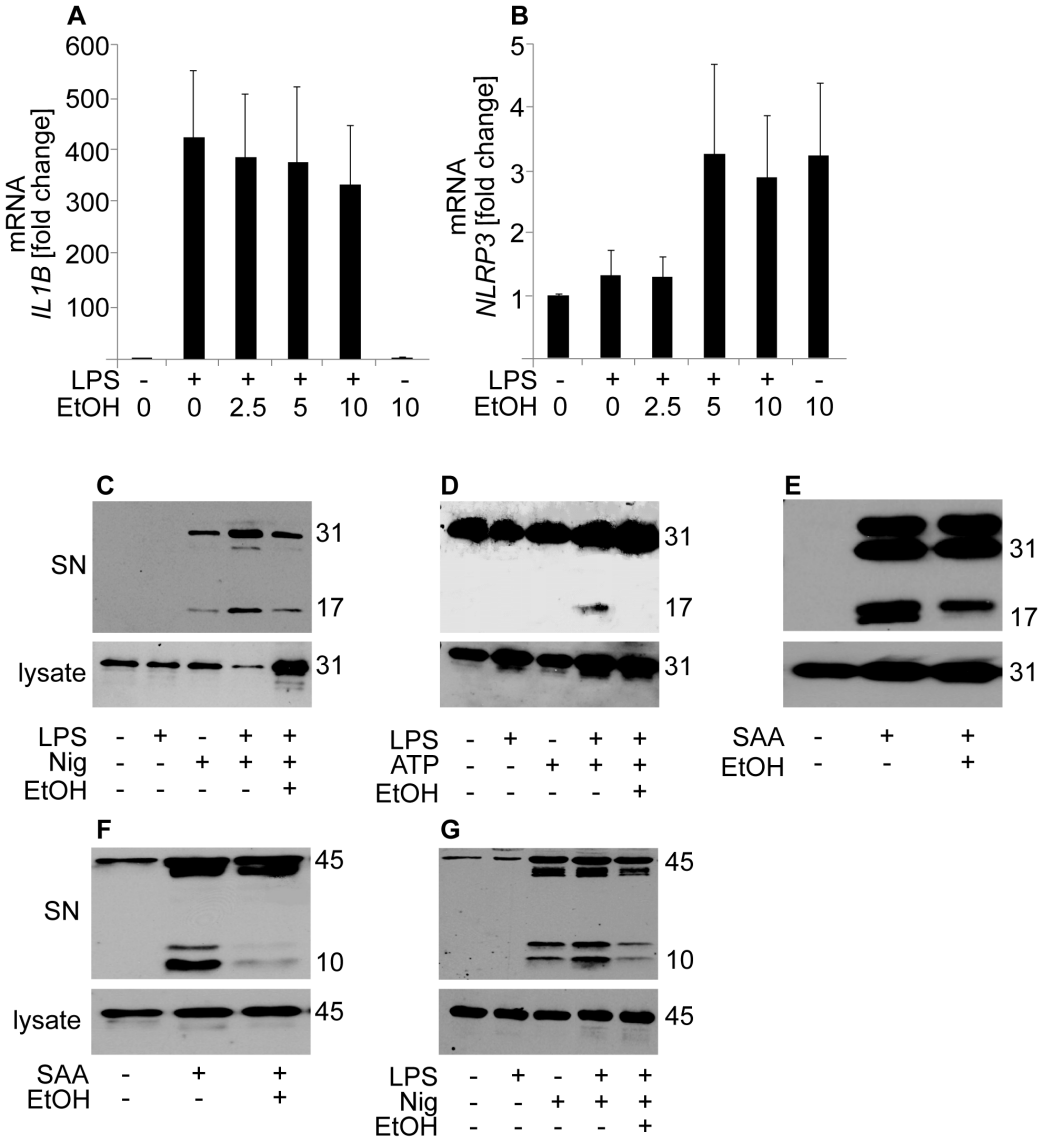
Ethanol inhibits the secretion of mature IL-1β and caspase-1, but not the expression of pro-IL-1β. For the measurement of mRNA expression, LPS-primed human primary macrophages were incubated in the presence of the indicated concentrations of ethanol and (**A**) *IL1B* and (**B**) *NLRP3* mRNA expression was determined by quantitative real-time RT-PCR. The data are expressed as fold changes compared to the control cells. The results are means ± s.e.m. from 5 individual experiments. For the Western blot studies LPS-primed human THP-1 cells were preincubated in 10‰ (171 mM) ethanol prior to the activation of the NLRP3 inflammasome with (**C,G**) nigericin and (**D**) ATP. For activation with (**E,F**) SAA no LPS priming was used. In IL-1β Western blots of supernatants (SN) or cell lysates (C–E), the p31 band represents pro-IL-1β and the p17 band mature IL-1β. In caspase-1 blots (F,G), the p45 band represents pro-caspase and the p10 band activated caspase-1. Results shown are representative of 3–4 individual experiments.

Statistical analyses were performed using GraphPad Prism version 6 for Windows. Differences between two groups were identified using the Student’s *t*-test for all analyses, except for the data in Figures 1A–B and 2A–B, which were analyzed using Wilcoxon matched-pairs signed rank test. Data are presented as means ± standard error of mean (s.e.m.) of data from three or more independent experiments. Statistical significance was set to p<0.05.

## Results

### Ethanol, but not Acetaldehyde, Inhibits Secretion of IL-1β

LPS-primed human primary macrophages or THP-1 cells were preincubated in the absence or presence of increasing concentrations of ethanol. Thereafter, an activator of the NLRP3 inflammasome, either cholesterol crystals, ATP, nigericin, or SAA was added, and the concentration of IL-1β in the culture medium was determined by ELISA. SAA induces the expression of pro-IL-1β through TLR2 and TLR4, therefore a separate priming signal (e.g. LPS) was not needed [31]. As shown in Fig. 1A–D, all the NLRP3 inflammasome activators strongly stimulated the release of IL-1β into the cell culture medium, while preincubation with ethanol dose-dependently inhibited the release. The above ELISA-assay results were further confirmed by Western blot analysis of the cell culture media, which specifically revealed an attenuation of secretion of the mature form of IL-1β (17 kDa) in the presence of ethanol (Fig. 2C–E and Fig. S1A–C). The inhibitory effect of ethanol on the secretion of IL-1β was evident also when ethanol was added simultaneously with ATP (Fig. S2A). In addition, ethanol inhibited the secretion of IL-18, another proinflammatory cytokine cleaved by caspase-1 (Fig. 1E–F). The amount of the secreted mature IL-18 protein was measured by ELISA. No ethanol-induced cytotoxicity was detected even at high ethanol concentrations; rather, ethanol slightly reduced cell death induced by the activation of the NLRP3 inflammasome (Fig. S3A–C).

In macrophages, ethanol can be further metabolized into acetaldehyde by CYP2E1 and, to a lesser extent, by alcohol dehydrogenase [36]. To study whether the inhibition of IL-1β secretion was caused by ethanol itself or by its metabolite, cells were treated with acetaldehyde. As demonstrated in Fig. S4A, even relatively high acetaldehyde concentrations (1 mM) had no significant effect on the ATP-induced secretion of IL-1β in the LPS-primed THP-1 cells. Furthermore, the inhibition of alcohol dehydrogenase with 4-methylpyrazole had no effect on the ethanol-mediated inhibition of the IL-1β secretion (Fig. S4B). These findings reveal that ethanol, but not acetaldehyde, is responsible for the observed inhibition of the IL-1β secretion.

### Ethanol Does Not Interfere with LPS-induced Priming of Macrophages

In primary human macrophages two separate signals are required for the secretion of mature IL-1β: first, priming of the cells via TLRs, which initiates the transcription of *IL1B* and *NLRP3*, and second, induction of the assembly of NLRP3 inflammasome and the activation of caspase-1, which then cleaves the pro-IL-1β into its mature secreted form. To study the effect of ethanol on the priming step, human primary macrophages were primed via TLR4 using LPS as ligand, and the mRNA levels of *IL1B* and *NLRP3* were determined. As shown in Fig. 2A, LPS strongly stimulated the expression of *IL1B* mRNA. Ethanol, when added after the LPS priming, did not reduce the expression of *IL1B.* No significant increase of *NLRP3* expression was observed after LPS stimulation (Fig. 2B). This finding accords with previous works demonstrating that upon stimulation with LPS the expression of *NLRP3* acutely rises and then rapidly returns to baseline [22], [37]. At the concentrations of 5‰ and 10‰ (corresponding to 86 mM and 171 mM) ethanol stimulated the expression of *NLRP3* (not statistically significant), which was observed also without LPS priming. These results suggest that the inhibitory effect of ethanol is not mediated via inhibition of the priming step but rather that ethanol inhibits the assembly and/or activation of the inflammasome.

Next we analyzed the effect of ethanol on the conversion of pro-IL-1β (31 kDa) into the mature IL-1β (17 kDa) and on its secretion from the cells. For this purpose, THP-1 cells were used since, after PMA differentiation, they stably express *IL1B* mRNA [38]. As shown in Fig. 2C–E, in total cell lysates pro-IL-1β protein was present without inflammasome activation. Preincubation of the cells in the presence of ethanol before the activation of the NLRP3 inflammasome did not reduce the cellular content of pro-IL-1β. However, in accordance with the results shown in Fig. 1, the amount of the secreted mature IL-1β in the culture medium was decreased in the presence of ethanol (Fig. 2C–E Fig. S1A–C). Taken together, the data presented above suggest that ethanol, when added after priming, does not significantly interfere with the expression of the pro-IL-1β in macrophages but, instead, inhibits the conversion of pro-IL-1β into its mature form.

### Ethanol Inhibits Activation of Caspase-1

Caspase-1 is a proenzyme (45 kDa) which, upon activation of the inflammasome, is autoproteolytically cleaved into active 10 kDa and 20 kDa forms. Active caspase-1 mediates the cleavage of pro-IL-1β into the mature secreted form. As demonstrated in Fig. 2F and G, both SAA and nigericin induced the secretion of caspase-1, and in particular the activated form of caspase-1 (10 kDa), from the THP-1 cells. Preincubation of the cells in the presence of 10‰ (171 mM) ethanol prior to the addition of nigericin or SAA resulted in clearly reduced levels of activated caspase-1 in the culture medium, while the level of intracellular procaspase-1 (a 45 kDa band) was not affected (Fig. 2F–G, Fig. S1D–E). These findings suggest that ethanol inhibits the autoproteolysis of caspase-1 by inhibiting the activation of inflammasome.

### Ethanol Inhibits Destabilization of Lysosomes and Leakage of Cathepsin B

To elucidate the mechanism(s) by which ethanol inhibits the activation of inflammasome we studied the effect of ethanol on the cellular processes involved in the activation of the NLRP3 inflammasome, i.e. lysosomal destabilization, leakage of cathepsin B, generation of ROS, and potassium efflux.

**Figure 3:**
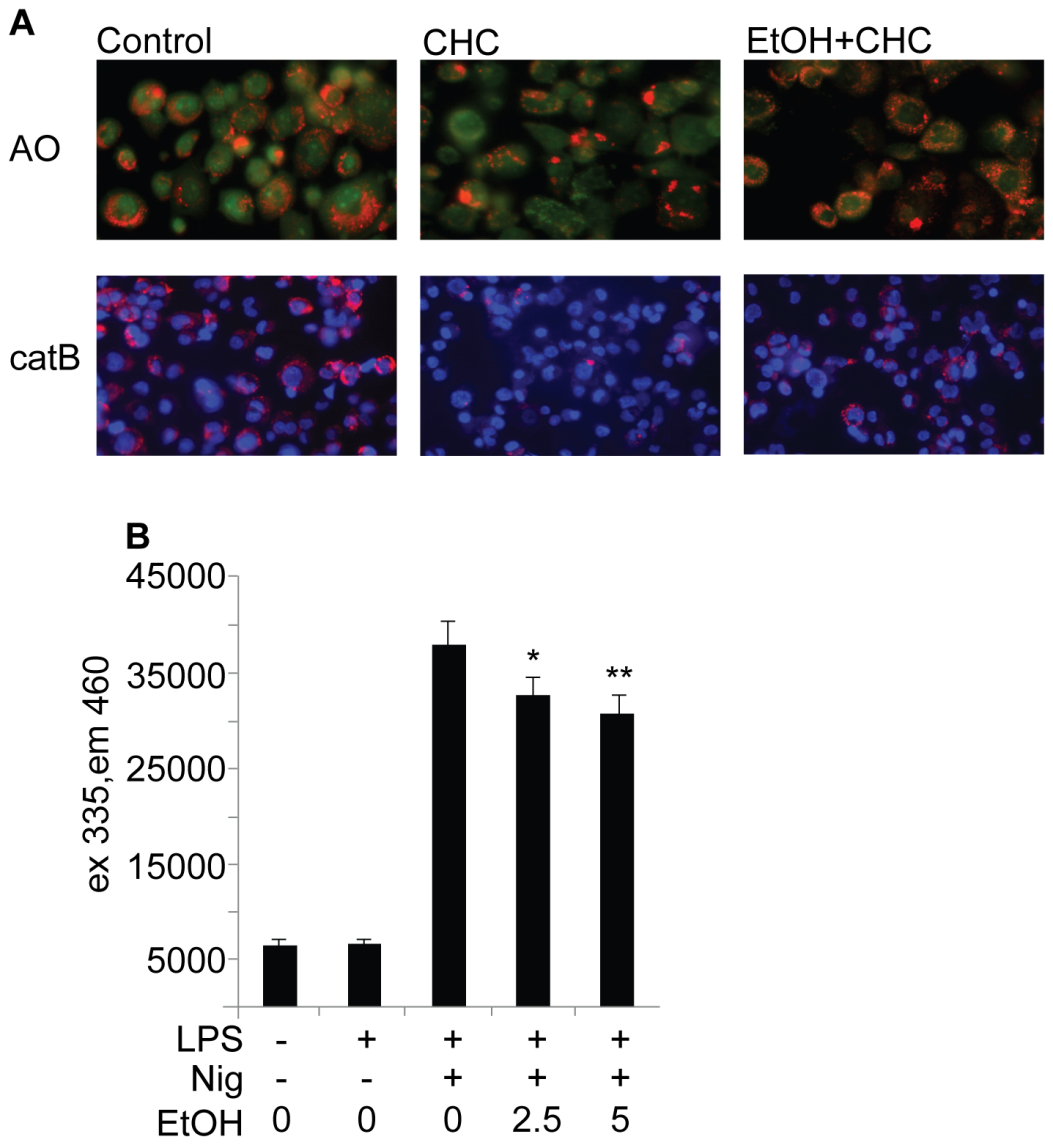
Ethanol inhibits lysosomal disruption and the secretion of active cathepsin B. (**A**) THP-1 cells were preincubated in 10‰ (171 mM) ethanol prior to the activation of the NLRP3 inflammasome with cholesterol crystals. Live cells were stained with acridine orange (AO), which changes color from green to orange/red in the acidic pH of lysosomes, or with fluorescently labeled cathepsin B substrate z-Arg-Arg-cresyl violet (catB), which emits red signal upon cleavage by cathepsin. The cells were imaged using epifluorescence microscopy. The images are representative of 3 experiments. (**B**) LPS-primed THP-1 cells were preincubated in the presence of indicated concentrations of ethanol prior to activation of the NLRP3 inflammasome with nigericin. Cathepsin B/L activity was determined from cell culture media using a fluorescently labeled substrate Z-Phe-Arg-AMC, and the fluorescence was measured after a 60 min incubation at +37°C. The results are expressed as means of ± s.e.m from 3 individual experiments performed in duplicate.

Lysosomal damage and the leakage of cathepsin B from lysosomes have been implicated as upstream events in the crystal-induced activation of the NLRP3 inflammasome [22], [23]. Acridine orange emits red fluorescence in acidic vacuoles, such as lysosomes, and the signal is lost when lysosomal integrity is compromised. As shown in Fig. 3A (upper panels), incubation of THP-1 cells with cholesterol crystals resulted in reduced acridine orange staining, indicating loss of lysosomal integrity. However, when the cells were treated with ethanol before the addition of cholesterol crystals, there was no clear attenuation of the acidic fluorescent signal, suggesting that ethanol reduces the cholesterol crystal-induced lysosomal destabilization. The protective effect was not due to an inhibition of the uptake of cholesterol crystals, as ethanol alone had no significant effect on the cholesterol crystal uptake of unstimulated (Fig. S5) or LPS-stimulated human primary macrophages (data not shown).

Cathepsin B is a lysosomal cysteine protease that is maximally active at the acidic pH normally present in lysosomes, but when released into a neutral milieu, such as the cytoplasm, cathepsin B becomes rapidly inactivated. To study whether ethanol affected the release of cathepsin B, THP-1 cells were stained with a cell-permeable cathepsin B substrate z-Arg-Arg-cresyl violet, which emits fluorescent signal when cleaved by cathepsin B [39], [40]. As shown in Fig. 3A (lower panels), cathepsin B activity (red) was observed in the unstimulated THP-1 cells. Upon addition of cholesterol crystals to the cells the staining was lost reflecting leakage of cathepsin B into the cytoplasm with ensuing inactivation. In line with the above-described studies with acridine orange, ethanol inhibited the cholesterol crystal-induced leakage of cathepsin B into the cytoplasm. In addition, the activity of cathepsin B in the cell culture media, as reflected by the cleavage of the cathepsin B/L substrate, was reduced in the presence of ethanol (Fig. 3B). These results suggest that ethanol attenuates the cholesterol crystal-induced lysosomal damage and the ensuing release of cathepsin B, as well as the nigericin-induced translocation of cathepsin B from intact lysosomes [33].

**Figure 4:**
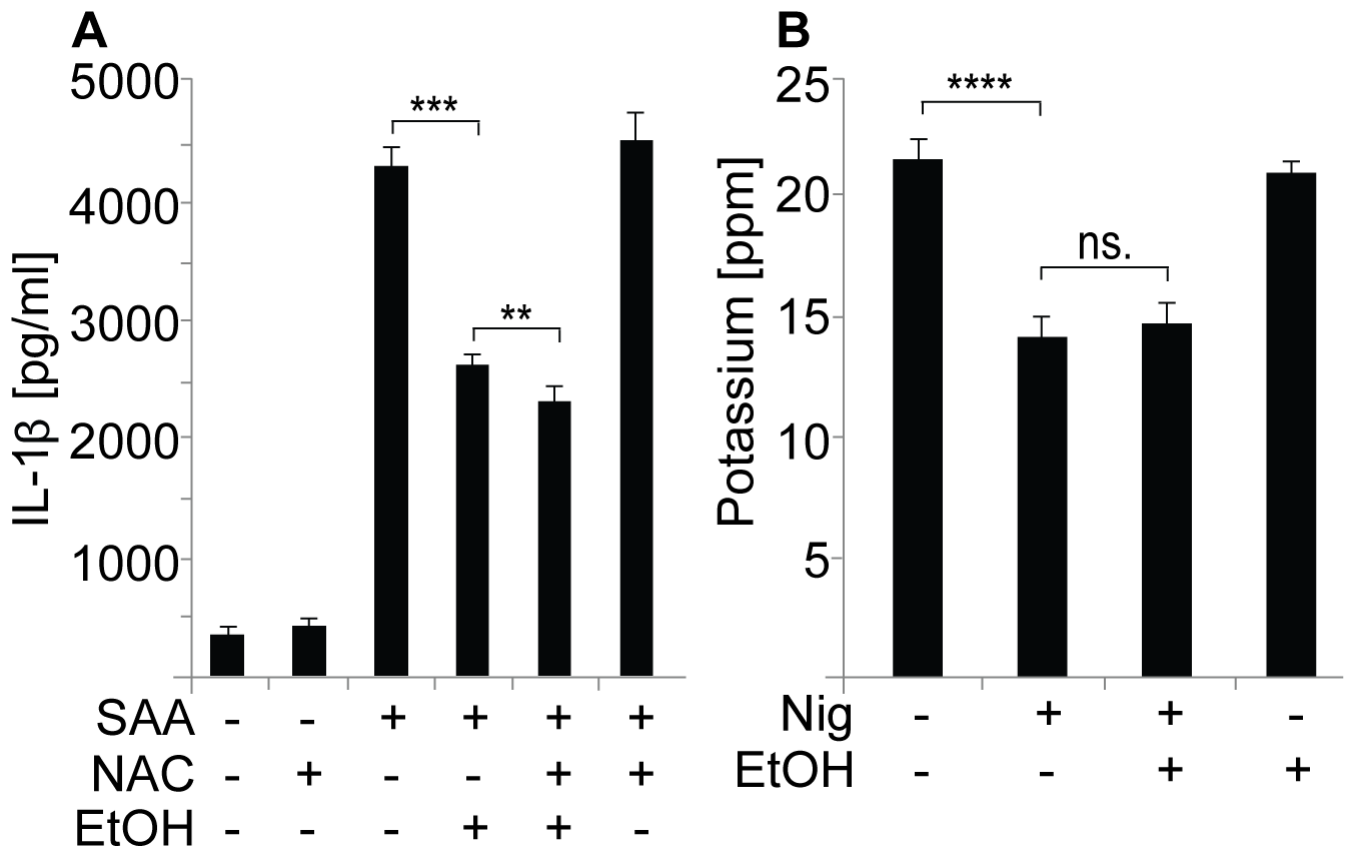
Potassium efflux and ROS are not involved in the ethanol-induced inhibition. (**A**) THP-1 cells were first preincubated with or without the ROS scavenger *N*-acetyl-L-cysteine (NAC), then in the presence of 10‰ (171 mM) ethanol, and the NLRP3 inflammasome was activated with SAA. Secretion of IL-1β into the culture medium was analyzed by ELISA. The results are expressed as the means ± s.e.m. from 4 individual experiments, performed in duplicate. (**B**) THP-1 cells were preincubated in 10‰ (171 mM) ethanol prior to activation of the NLRP3 inflammasome with nigericin. Intracellular potassium was determined by ICP-MS measurement of cell lysates. The results are expressed as means ± s.e.m. from 3 individual experiments.

Another mechanism proposed to mediate the activation of the NLRP3 inflammasome is the enhanced formation of intracellular ROS [20], [41]. However, the role of ROS has been challenged [42], [43] and the overproduction of ROS has been shown also to inhibit the activation of caspase-1 [44]. Since ethanol treatment increases oxidative stress and induces the generation of ROS in several cell types [45], [46], we examined whether the enhanced ROS production, induced by ethanol, could contribute to the inhibitory effect of ethanol. When added to THP-1 cells, the general ROS inhibitor *N*-acetyl-L-cysteine (NAC) had a clear inhibitory effect on the ATP-induced activation of the NLRP3 inflammasome (Fig. S6) but had no effect on the SAA-induced activation of the NLRP3 inflammasome (Fig. 4A). Importantly, NAC did not reverse the ethanol-mediated inhibition of the SAA-induced (Fig. 4A) or ATP-induced (Fig. S6) secretion of IL-1β. This suggests that ethanol-induced enhancement of ROS generation does not contribute to the inhibition of IL-1β secretion in THP-1 cells.

Depletion of intracellular potassium is involved in the initiation of the second step in the ATP and nigericin-induced activation of the NLRP3 inflammasome [47]. To study whether ethanol interferes with potassium efflux, intracellular potassium concentrations were measured in THP-1 cells by using inductively coupled plasma mass spectrometry (ICP-MS). As demonstrated in Fig. 4B, activation of THP-1 cells with nigericin resulted in significantly lower intracellular potassium levels. Yet, pretreatment of the cells with ethanol did not have any effect on the intracellular potassium concentration (Fig. 4B).

### Ethanol Inhibits Secretion and Oligomerization of ASC

**Figure 5:**
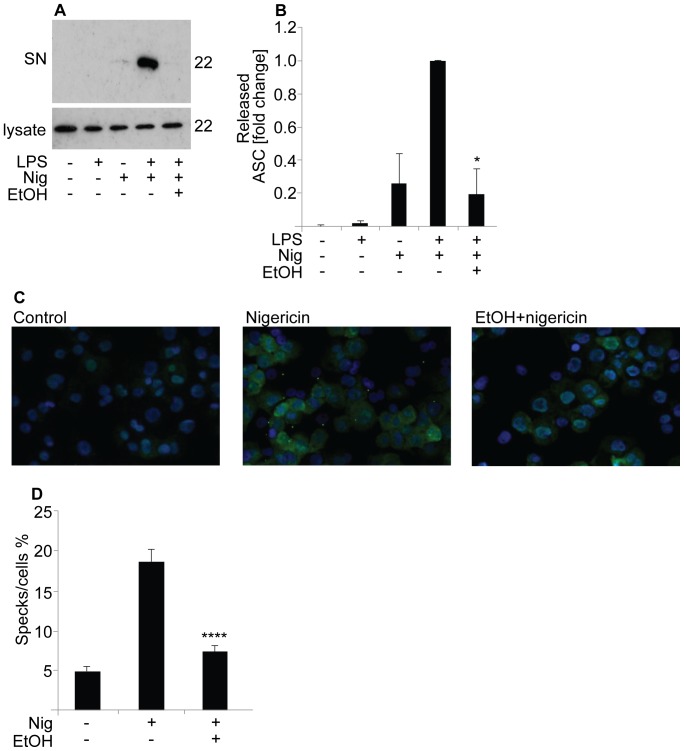
Ethanol inhibits release and oligomerization of ASC. (**A**) LPS-primed THP-1 cells were preincubated in 10‰ (171 mM) ethanol prior to the activation of the NLRP3 inflammasome with nigericin. ASC (22 kDa) was blotted from the supernatants (SN) and cell lysates. Western blots shown are representative of 4 experiments. (**B**) The intensities of the ASC bands of the cell culture supernatants were analyzed from 4 individual experiments, and are expressed as fold changes compared to the LPS-primed nigericin-activated cells. The results are expressed as means of ± s.e.m. (**C**) THP-1 cells were preincubated in 10‰ (171 mM) ethanol prior to the activation of the NLRP3 inflammasome with nigericin. Cells were fixed and analyzed for ASC expression using epifluorescence microscopy. (**D**) To quantify the extent of speck formation, the number of specks was divided by the number of cells per field and expressed as a percentage. The results are expressed as means ± s.e.m. from 4 individual experiments.

ASC is a key protein mediating the interaction between NLRP3 and caspase-1. Upon activation of the inflammasome, ASC is secreted from the cells along with other inflammasome components [48]. As demonstrated in Fig. 5A–B, activation of LPS-primed THP-1 cells with nigericin resulted in marked secretion of ASC (22 kDa) into the culture medium. Interestingly, pretreatment of the cells with ethanol resulted in strong inhibition of ASC secretion. Conversely, ethanol had no effect on the expression of the ASC protein. This suggests that ethanol interferes with the assembly of the inflammasome and/or with the secretion of the inflammasome components. To further study the effect of ethanol on the assembly of the NLRP3 inflammasome, THP-1 cells were stained with a monoclonal antibody against ASC. One hour after activation with nigericin the oligomerization of ASC, as reflected by the formation of ASC specks, was monitored with epifluorescence microscopy (Fig. 5C). A clear induction in the formation of ASC specks was observed in the nigericin-treated cells, however, preincubation of the cells with ethanol significantly prevented the nigericin-dependent increase in the formation of ASC specks (Fig. 5D).

### Ethanol Reduces the AIM2 Inflammasome-mediated Secretion of IL-1β

**Figure 6:**
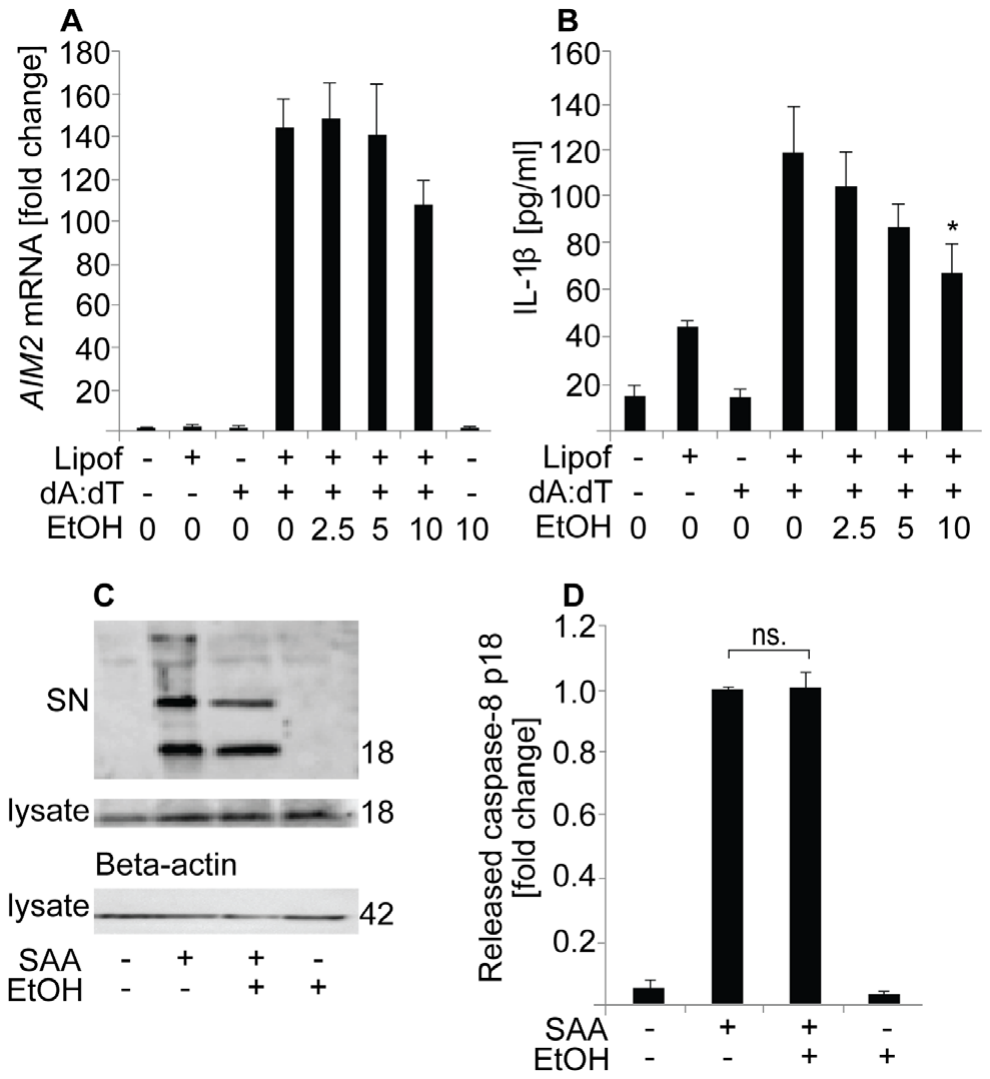
Ethanol inhibits AIM2 inflammasome-induced secretion of IL-1β, but not activation of caspase-8. (**A**) For the measurement of *AIM2* mRNA expression THP-1 cells were preincubated in the presence of the indicated concentrations of ethanol prior to the activation of the AIM2 inflammasome by transfecting the cells with poly (dA:dT). The results are expressed as fold changes compared to unstimulated cells. The data are expressed as means ± s.e.m. from 4 individual experiments. (**B**) To study the activation of the AIM2 inflammasome, THP-1 cells were preincubated in the presence of the indicated concentrations of ethanol and thereafter the AIM2 inflammasome was activated by transfecting the cells with poly (dA:dT), and the secretion of IL-1β into the culture medium was determined by ELISA. The results are expressed as means ± s.e.m. from 5 individual experiments. (**C**) THP-1 cells were preincubated in the presence of 10‰ (171 mM) ethanol prior to the activation of the cells with SAA. The activated caspase-8 (p18) was detected from the supernatants and cell lysates. Beta-actin is shown as a loading control. (**D**) The intensities of the activated caspase-8 bands of the cell culture supernatants were analyzed from 4 individual experiments, and are expressed as fold changes compared to the activated cells. The results are expressed as means of ± s.e.m.

To study whether the inhibitory effect of ethanol is specific for the NLRP3 inflammasome, we tested the ability of ethanol to inhibit also the function of the absent in melanoma 2 (AIM2) inflammasome. To assess this, we transfected THP-1 cells with synthetic dsDNA analog [Poly (dA:dT)] and the production of IL-1β was measured. As demonstrated in Fig. 6A, ethanol did not inhibit the *AIM2* mRNA expression, but it inhibited the activation of the AIM2 inflammasome as reflected by dose dependent reduction of IL-1β secretion (Fig. 6B). Thus, the inhibitory effect of ethanol appears not to be restricted to the NLRP3 inflammasome.

### Ethanol Does Not Interfere with Activation of Caspase-8

Recently, activated caspase-8 has also been shown to cleave IL-1β within a complex that is formed in response to the activation of dectin-1 receptor and inflammasome [49], [50]. Unlike caspase-1, caspase-8 interacts with the pyrin domain of ASC [50], [51]. Interestingly, ethanol blocked the SAA and nigericin-induced activation of caspase-1 (Fig. 2F–G and Fig. S1D–E), but had no significant effect on the SAA-induced activation of caspase-8 (Fig. 6C–D).

## Discussion

In the present study we show that in human macrophages ethanol dose-dependently inhibits the processing and release of the two NLRP3-dependent cytokines, IL-1β and IL-18. Ethanol inhibited the activation of the NLRP3 inflammasome by cholesterol crystals, SAA, ATP, and nigericin over a wide range of concentrations (2.5‰–20‰ corresponding to 43 mM–343 mM). The lowest concentrations of ethanol used are comparable to those observed in tissues *in vivo* after ingestion of ethanol. After oral ingestion of alcohol the cells of the upper gastrointestinal tract and gastric mucosa are exposed to clearly higher ethanol concentrations than those in blood and extracellular fluids, and these concentrations are comparable to the highest concentrations used in the study. Furthermore, the ethanol concentrations used, did not affect the viability of the macrophages (Fig. S3).

**Figure 7:**
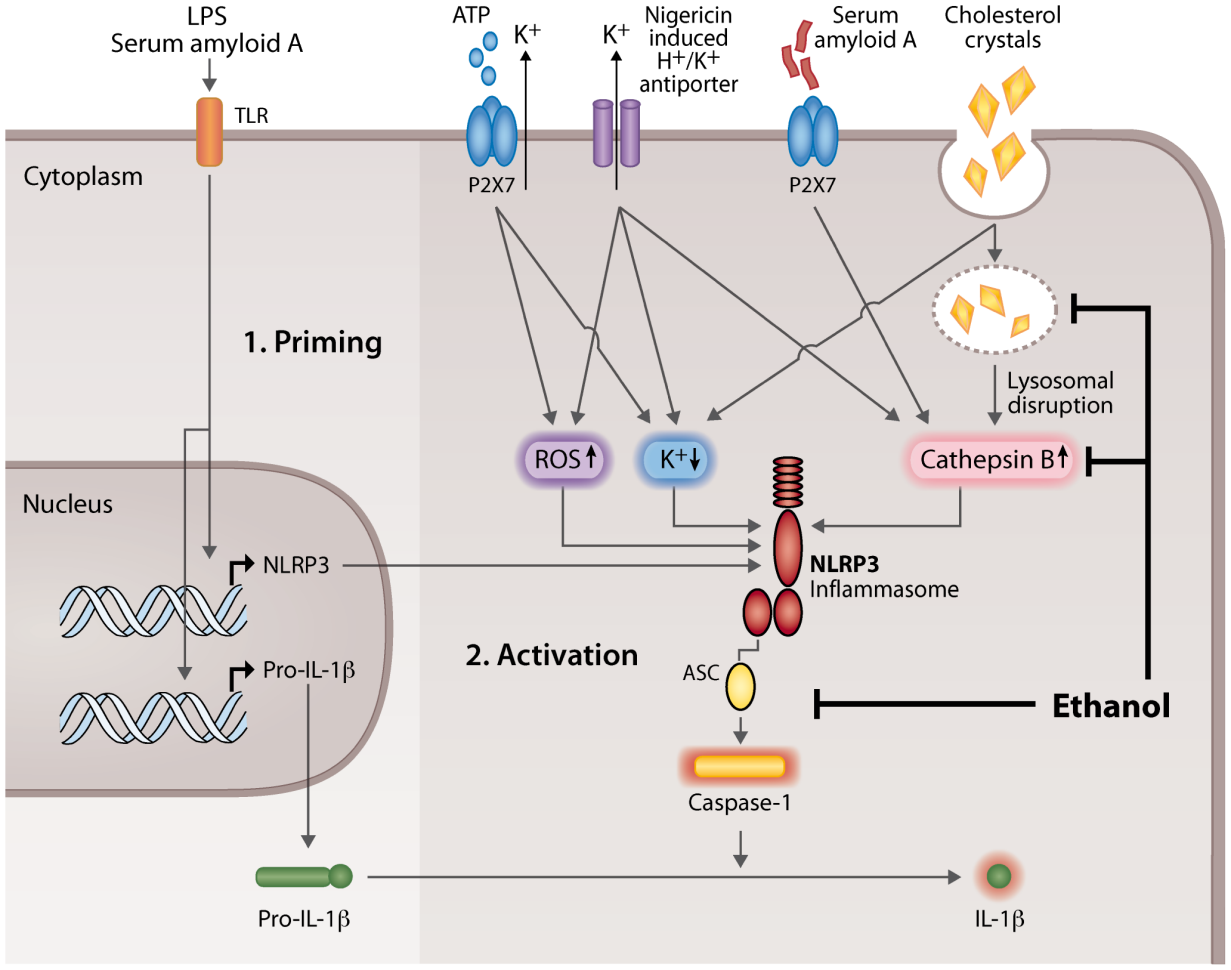
The effect of ethanol on the NLRP3 inflammasome-activating danger signals. 1. Priming (left). Binding of lipopolysaccharide (LPS) or serum amyloid A to a toll-like receptor (TLR) activates the receptor which then initiates the synthesis of NLRP3 (nucleotide-binding domain and leucine-rich repeat containing family, pyrin domain containing 3) and pro-interleukin-1β (pro-IL-1β). 2. Activation (right). The pathways leading to activation of NLRP3 inflammasome by the 4 agents (ATP, nigericin, serum amyloid A, and cholesterol crystals) used in this study are shown. Extracellular ATP binds to the P2X purinergic receptor 7 (P2X7), so inducing potassium efflux and reciprocal lowering of the intracellular potassium concentration. Likewise, nigericin induces K^+^ efflux through its H+/K+ antiporter function and ensuing lowering of the intracellular potassium concentration. Finally, also cholesterol crystals lower the concentration of intracellular potassium. Both ATP and nigericin also trigger the formation of reactive oxygen species (ROS). Nigericin and serum amyloid A, the latter by activating the P2X7 receptor, induce the release of cathepsin B from intact lysosomes into the cytoplasm (intact lysosomes not shown for clarity). Phagocytosed cholesterol crystals cause lysosomal disruption and the leakage of cathepsin B into the cytoplasm. The cytoplasmic elevation of ROS, lowering of potassium, and the elevation of active cathepsin B, each can activate the NLRP3 inflammasome. The activated NLRP3 inflammasome contains active enzyme caspase-1, which cleaves pro-IL-1β and so converts it into the active secretable form. Ethanol inhibits the activation of the NLRP3 inflammasome, thereby reducing the activation of caspase-1 and the secretion of mature IL-1β at two levels: 1) by inhibiting the release of cathepsin B from both the intact and disrupted lysosomes, and 2) by diminishing the assembly of the NLRP3 inflammasome complexes.

Priming is necessary for the secretion of mature IL-1β in macrophages. Inflammatory stimuli, such as activation of TLRs, induce the priming of the NLRP3 inflammasome via activation of nuclear factor-κB (NF-κB), which results in the induction of *IL1B* and *NLRP3* mRNA expression [37]. Previous studies have shown that ethanol, when administered prior to a TLR receptor activator, inhibits the expression of proinflammatory cytokines in cells of the monocyte-macrophage lineage, by inhibiting the MAPK (mitogen-activated protein kinase) and NF-κB pathways [52], [53]. However, when ethanol is administered simultaneously with the TLR2 and TLR4 activators, ethanol rather augments the NF-κB activation, JNK (c-Jun N-terminal kinase) phosphorylation, and AP-1 (activator protein 1) nuclear binding [54]. In the present study the cells were first primed with a TLR4 receptor agonist (LPS) and ethanol was added thereafter. Under these conditions ethanol did not significantly affect the mRNA expression of *IL1B*, and in accordance with this observation, ethanol did not affect the level of intracellular pro-IL-1β protein (Fig. 2C–E). Ethanol also did not reduce the expression of the *NLRP3*, but somewhat increased its expression (not statistically significant). Altogether, these data suggest that the observed reduction in the secretion of mature IL-1β and IL-18 by ethanol is not caused by an inhibition of TLR-mediated priming of the NLRP3 inflammasome, but instead by the inhibition of the NLRP3 inflammasome activation (Fig. 7). Interestingly, ethanol also inhibited the AIM2 inflammasome-induced secretion of IL-1β. The AIM2 inflammasome is a member of PYHIN (pyrin and HIN200 domain containing) protein family, which is activated by a broad range of double-stranded DNA from viral, bacterial, mammalian, or synthetic sources [25]. Thus, the inhibitory effect of ethanol was not restricted to the NLRP3-dependent secretion of IL-1β by human macrophages.

The initiating mechanisms leading to the assembly and activation of the NLRP3 inflammasome are not fully understood. Enhanced generation of ROS, potassium efflux, and destabilization of lysosomes have all been implicated as triggering mechanisms. ROS is generated under both physiological and pathological conditions, but elevation of ROS does not always lead to the activation of the inflammasome. The role of ROS has also been challenged by findings that cells deficient of NADPH oxidase activity show increased activation of the NLRP3 inflammasome [42], [43]. Furthermore, overproduction of ROS has been shown also to inhibit the inflammasome through oxidation and glutathionylation of caspase-1 [44]. Ethanol metabolism has been shown to induce oxidative stress and ROS production [46], [55]. Therefore, the role of the ethanol-induced ROS generation in the inhibition of the inflammasome activation was assessed. However, inhibition of ROS formation did not reduce the ability of ethanol to inhibit the inflammasome (Fig. 4A), suggesting that the ethanol-induced generation of ROS is not significantly involved in the inhibitory effect.

Ethanol did not have any significant effect on the potassium efflux induced by nigericin (Fig. 4B), demonstrating that ethanol does not inhibit the H+/K+ antiporter function of this potassium ionophore. It also seems unlikely that the ethanol-mediated inhibition of the NLRP3 inflammasome was due to modulation of ATP receptor P2X_7_, as ethanol inhibited also nigericin-induced activation of the inflammasome, which is independent of the P2X_7_ receptor function.

Lysosomal damage and the subsequent leakage of cathepsin B have been implicated in the activation of the NLRP3 inflammasome by cholesterol crystals [22], [23]. In the present study ethanol significantly reduced the cholesterol crystal-induced secretion of IL-1β, and moreover, ethanol also inhibited lysosomal disruption, and the release of cathepsin B (Fig. 3A–B). These data indicate that the inhibitory effect of ethanol on cholesterol crystal-induced inflammasome activation is mediated via stabilization of lysosomes and/or by inhibition of intracellular events upstream of the activation of the NLRP3 inflammasome.

ASC is a key adaptor protein, required by most inflammasome receptors to mediate the caspase-1 activation, and consequently, the inhibition or deletion of ASC results in the inhibition of caspase-1 activation [17], [56]. Upon inflammasome activation, ASC oligomerizes and large intracellular oligomeric structures called ASC specks are formed. Inflammasome activation also results in the secretion of ASC from the cells along with other inflammasome components [48]. Here were demonstrated that ethanol significantly inhibited the secretion of ASC in nigericin-activated macrophages (Fig. 5A–B). Furthermore, in the presence of ethanol the oligomerization of ASC, i.e. the formation of ASC specks, was almost fully inhibited (Fig. 5C-D). Inhibition of ASC oligomerization and recruitment to the NLRP3 inflammasome complexes has been previously described as a mode of action of certain inhibitors of IL-1β secretion, including cytokine release inhibitory drugs, pyrin domain-only proteins and inhibitors of the deubiquitinase enzyme [57], [58], [59]. Since ASC is a critical factor for the formation of the both NLRP3 and AIM2 inflammasomes, the observed inhibition of ASC oligomerization by ethanol may represent the key mechanism explaining the ability of ethanol to inhibit both inflammasomes in macrophages. The activation of the recently discovered caspase-8 inflammasome is also crucially dependent of ASC, but the interaction between caspase-8 and ASC is mediated via the pyrin domain of ASC [50], [51] instead of the CARD (caspase activating and recruitment domain) that recruits caspase-1 [24], [25]. Intriguingly, ethanol was not able to inhibit the activation of caspase-8 induced by SAA in THP-1 cells (Fig. 6C–D), which implicates that ethanol is able to disturb the caspase interaction with the CARD domain but not with the pyrin domain of ASC. However, the exact mechanism by which ethanol inhibits the ASC oligomerization remains to be elucidated.

The activation of the inflammasomes and the consequent secretion of proinflammatory cytokines IL-1β and IL-18 is an important defense mechanism of innate immunity. Accordingly, the inhibition of the NLRP3 and AIM2 inflammasomes by ethanol may contribute to the increased incidence of bacterial infections associated with excessive use of alcohol [60], [61]. Notably, the activation of the NLRP3 inflammasome has been recently implicated also in the pathogenesis of atherosclerosis [22], [23]. Previously the ethanol-induced increase of HDL has been suggested to play a major role in reducing the risk of CAD among moderate drinkers. Recent studies, however, show that drugs that increase the level of circulating HDL cholesterol do not decrease the risk of cardiovascular diseases [62]. This suggests that the anti-inflammatory effects of ethanol may be more important for its atheroprotective activity. In particular, inhibition of the cholesterol crystal-induced activation of the NLRP3 inflammasome in macrophages of atherosclerotic lesions may represent a novel mechanism in the atheroprotective effect of moderate alcohol consumption.

### Conclusions

Ethanol inhibits the secretion of IL-1β and IL-18 in cultured human macrophages by inhibiting the activation of the NLRP3 and AIM2 inflammasomes. The inhibition of the inflammasomes by alcohol may contribute to the propensity for infections associated with excessive use of alcohol, and to the atheroprotective effect of moderate alcohol consumption.

## Supporting Information

Figure S1 **Ethanol inhibits the activation of IL-1β and caspase-1.** Quantitation of the Western blots of which representative blots are shown in Figure 2. LPS-primed THP-1 cells were preincubated in the presence of 10‰ (171 mM) ethanol prior to the activation of the NLRP3 inflammasome with **(A,E)** nigericin, **(B)** ATP or **(C,D)** SAA. For activation with SAA no LPS priming was used. The active forms of IL-1β (p17) and caspase-1 (p10) were detected from the supernatants by Western blotting. The intensities of the bands were analyzed from 3 (B) and 4 (A,C-E) individual experiments, and are expressed as fold changes compared to the activated cells. The results are expressed as means of ± s.e.m.
(TIF)

Figure S2 **The effect of ethanol is immediate and is not fully reversed after its removal. (A)** Ethanol (final concentration 10‰ corresponding to 171 mM) was added simultaneously with ATP to LPS-primed human primary macrophages. The results are expressed as means ± s.e.m from 5 individual experiments, performed in duplicate. **(B)** LPS-primed human primary macrophages were preincubated in the presence of 10‰ (171 mM) ethanol for 3 h and then activated with ATP 10 min (black bars) or 60 min (grey bars) after the removal of ethanol. The results are expressed as means ± s.e.m. from 8 individual experiments, performed in triplicate. Secretion of IL-1β into the culture medium (A–B) was analyzed by ELISA.
(TIF)

Figure S3 **Ethanol has no effect on cell death induced by inflammasome activation.** Human macrophages were preincubated in the presence of indicated concentrations of ethanol prior to the activation of the NLRP3 inflammasome with **(A)** ATP, **(B)** nigericin and **(C)** SAA. LPS-priming was used only in ATP activation. The release of lactate dehydrogenase was measured from the culture media of either human primary macrophages (A) or THP-1 cells (B,C). The data are expressed as cytotoxicity %, according to manufactureŕs recommendations. The results are expressed as means ± s.e.m from 3–4 individual experiments.
(TIF)

Figure S4 **Acetaldehyde has no effect on the ATP-induced secretion of IL-1β. (A)** LPS-primed THP-1 cells were preincubated in the presence of the indicated concentrations (given in µM) of acetaldehyde (AA) for 3 hours prior to the activation of the NLRP3 inflammasome with ATP. The results are expressed as the means ± s.e.m. from 4 individual experiments. **(B)** LPS-primed human primary macrophages were preincubated with or without the alcohol dehydrogenase inhibiting compound 4-methylpyrazole (4-MP: 2 h, 1 µM), after which ethanol (final concentration 10‰ corresponding to 171 mM) was added, and then NLRP3 inflammasome was activated with ATP. The data are expressed as fold changes compared to the LPS-primed ATP activated cells. The results are expressed as means ± s.e.m from 3 individual experiments. Secretion of IL-1β into the culture medium (A–B) was analyzed by ELISA.
(TIF)

Figure S5 **Ethanol has no effect on phagocytosis of cholesterol crystals by macrophages.** Human primary macrophages were preincubated in the presence of the indicated concentrations of ethanol prior to addition of cholesterol crystals. Cellular cholesterol uptake was analyzed by thin layer chromatography by measuring cellular content of cholesteryl esters (CE). The data are expressed as per cent changes compared to macrophages incubated with cholesterol crystals in the absence of ethanol. The results are expressed as means ± s.e.m from 3 individual experiments.
(TIF)

Figure S6 **Scavenging reactive oxygen species do not influence the inhibitory effect of ethanol.** LPS-primed THP-1 cells were first preincubated with or without the ROS scavenger *N*-acetyl-L-cysteine (NAC) then ethanol (final concentration 10‰ corresponding to 171 mM) was added, and finally the NLRP3 inflammasome was activated with ATP. Secretion of IL-1β into the culture medium was analyzed by ELISA. The results are expressed as means ± s.e.m. from 4 individual experiments, performed in duplicate.
(TIF)

Table S1 **Primers and probes for quantitative real-time RT-PCR.**
(DOCX)
