# Supplementary figures and images for: Ethanol Inhibits Activation of NLRP3 and AIM2 Inflammasomes in Human Macrophages–A Novel Anti-Inflammatory Action of Alcohol
Source: PLoS One. 2013 Nov 11;8(11):e78537. doi: 10.1371/journal.pone.0078537 (PMC3823849; doi:10.1371/journal.pone.0078537)

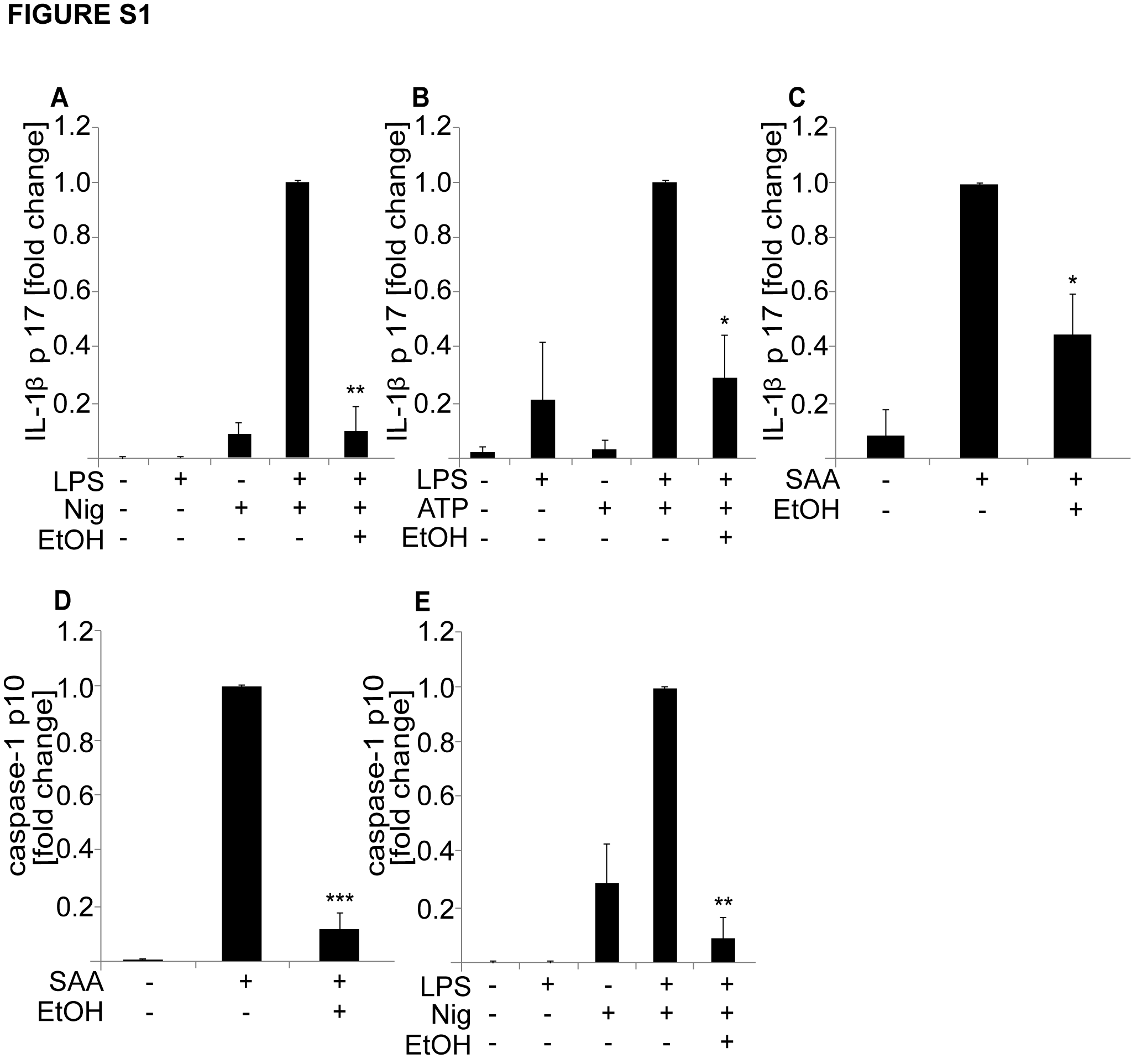

Supplement: Figure S1 — Ethanol inhibits the activation of IL-1β and caspase-1. Quantitation of the Western blots of which representative blots are shown in Figure 2. LPS-primed THP-1 cells were preincubated in the presence of 10‰ (171 mM) ethanol prior to the activation of the NLRP3 inflammasome with (A,E) nigericin, (B) ATP or (C,D) SAA. For activation with SAA no LPS priming was used. The active forms of IL-1β (p17) and caspase-1 (p10) were detected from the supernatants by Western blotting. The intensities of the bands were analyzed from 3 (B) and 4 (A,C-E) individual experiments, and are expressed as fold changes compared to the activated cells. The results are expressed as means of ± s.e.m. (TIF) [file pone.0078537.s001.tif]

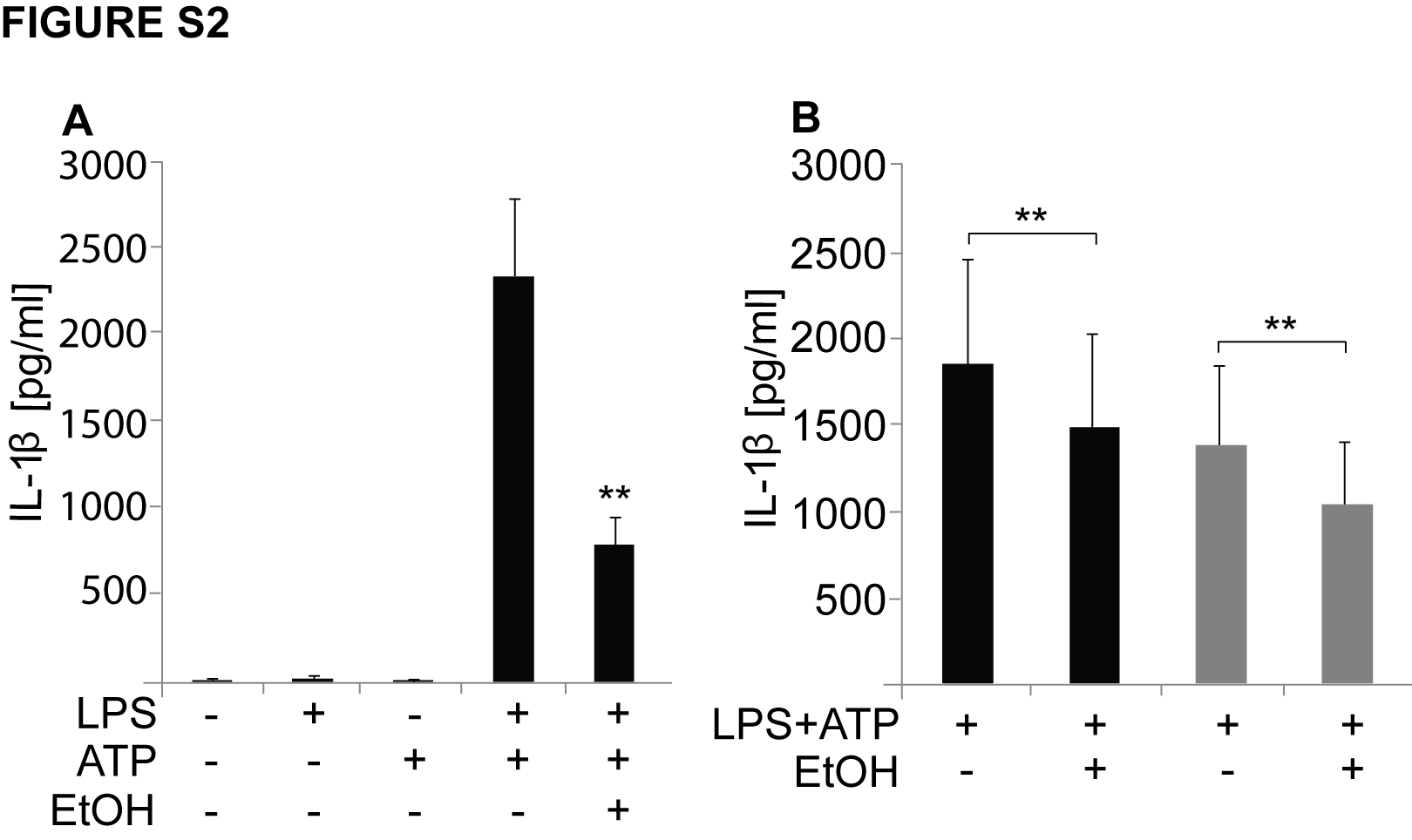

Supplement: Figure S2 — The effect of ethanol is immediate and is not fully reversed after its removal. (A) Ethanol (final concentration 10‰ corresponding to 171 mM) was added simultaneously with ATP to LPS-primed human primary macrophages. The results are expressed as means ± s.e.m from 5 individual experiments, performed in duplicate. (B) LPS-primed human primary macrophages were preincubated in the presence of 10‰ (171 mM) ethanol for 3 h and then activated with ATP 10 min (black bars) or 60 min (grey bars) after the removal of ethanol. The results are expressed as means ± s.e.m. from 8 individual experiments, performed in triplicate. Secretion of IL-1β into the culture medium (A–B) was analyzed by ELISA. (TIF) [file pone.0078537.s002.tif]

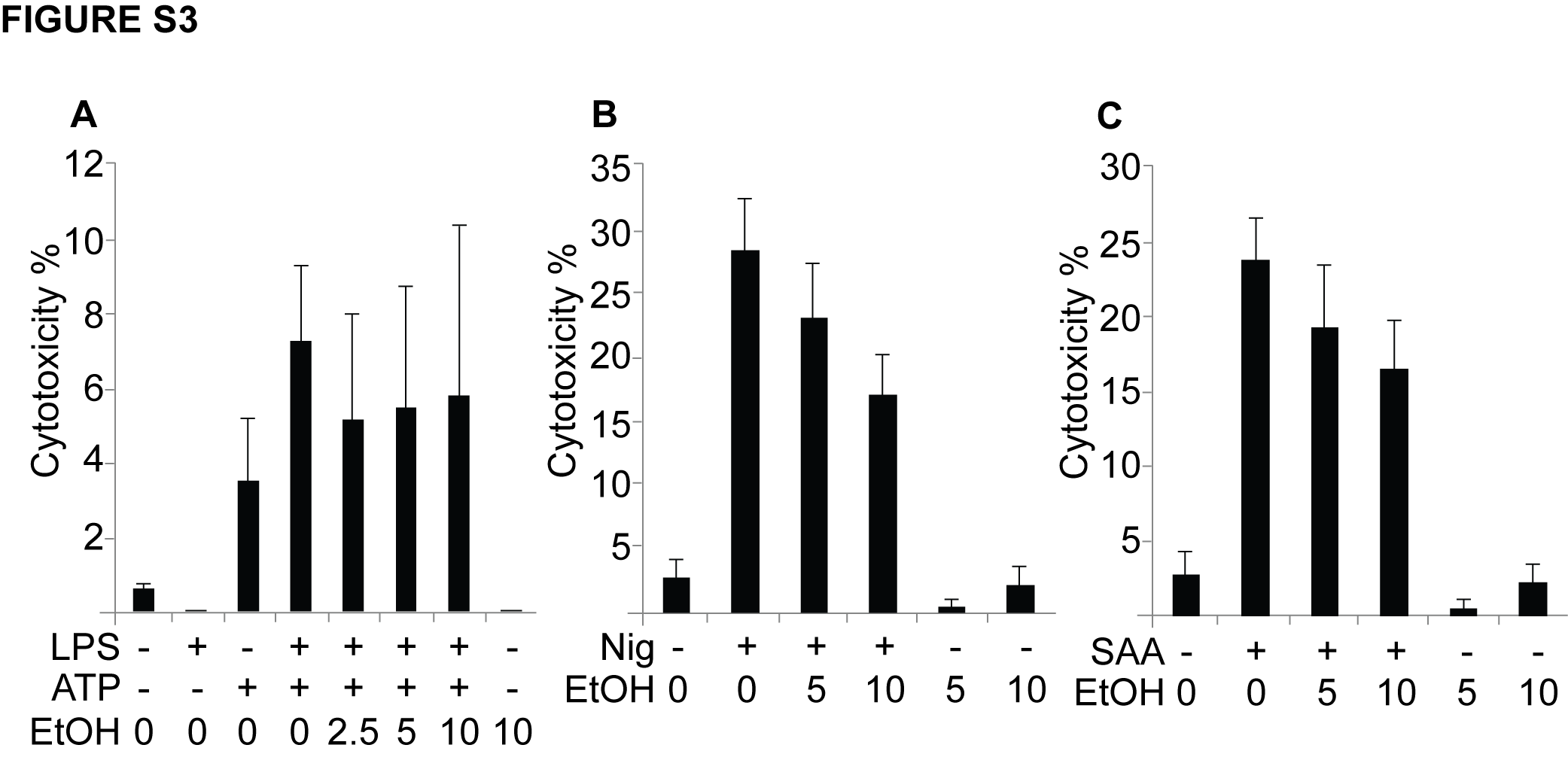

Supplement: Figure S3 — Ethanol has no effect on cell death induced by inflammasome activation. Human macrophages were preincubated in the presence of indicated concentrations of ethanol prior to the activation of the NLRP3 inflammasome with (A) ATP, (B) nigericin and (C) SAA. LPS-priming was used only in ATP activation. The release of lactate dehydrogenase was measured from the culture media of either human primary macrophages (A) or THP-1 cells (B,C). The data are expressed as cytotoxicity %, according to manufactureŕs recommendations. The results are expressed as means ± s.e.m from 3–4 individual experiments. (TIF) [file pone.0078537.s003.tif]

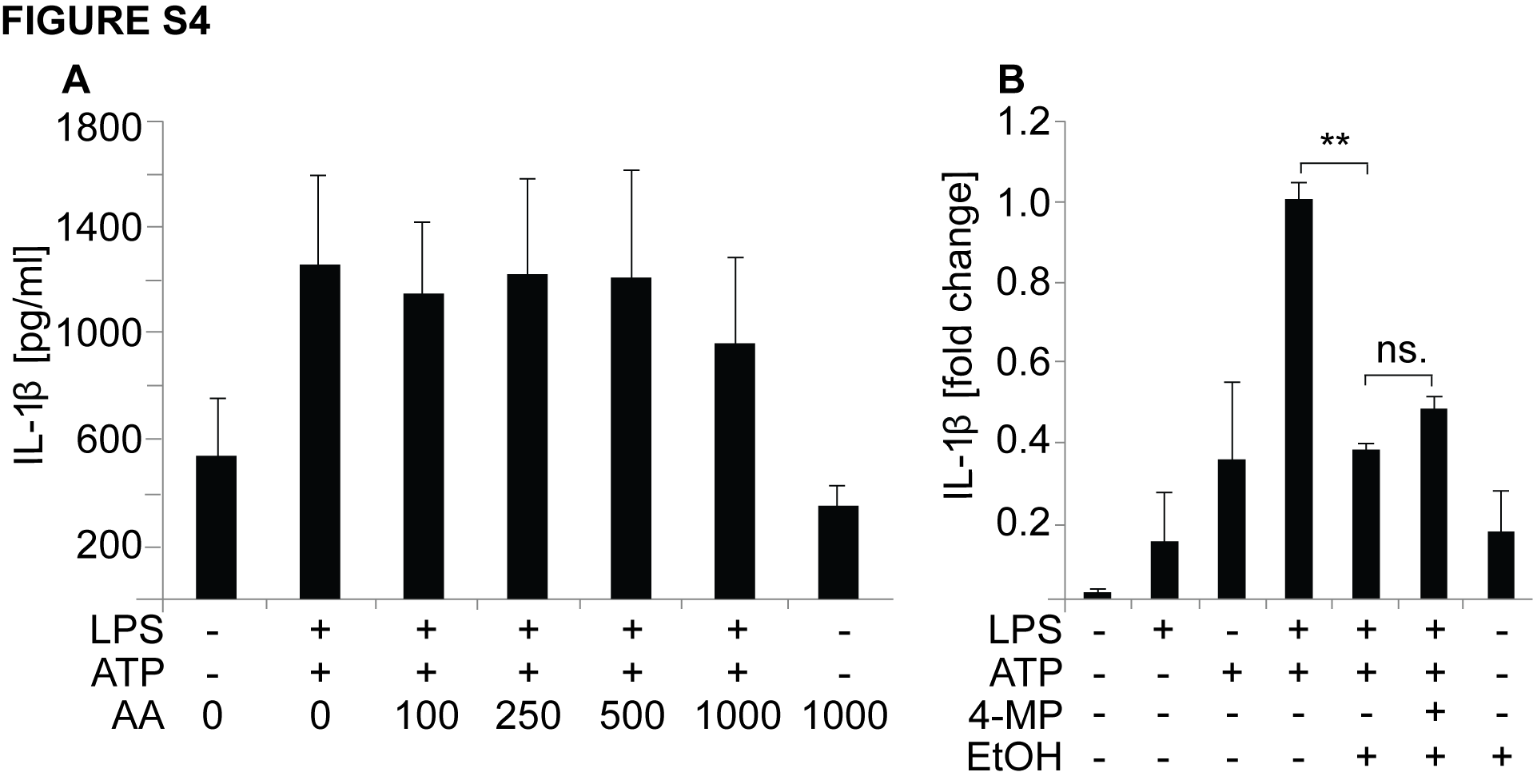

Supplement: Figure S4 — Acetaldehyde has no effect on the ATP-induced secretion of IL-1β. (A) LPS-primed THP-1 cells were preincubated in the presence of the indicated concentrations (given in µM) of acetaldehyde (AA) for 3 hours prior to the activation of the NLRP3 inflammasome with ATP. The results are expressed as the means ± s.e.m. from 4 individual experiments. (B) LPS-primed human primary macrophages were preincubated with or without the alcohol dehydrogenase inhibiting compound 4-methylpyrazole (4-MP: 2 h, 1 µM), after which ethanol (final concentration 10‰ corresponding to 171 mM) was added, and then NLRP3 inflammasome was activated with ATP. The data are expressed as fold changes compared to the LPS-primed ATP activated cells. The results are expressed as means ± s.e.m from 3 individual experiments. Secretion of IL-1β into the culture medium (A–B) was analyzed by ELISA. (TIF) [file pone.0078537.s004.tif]

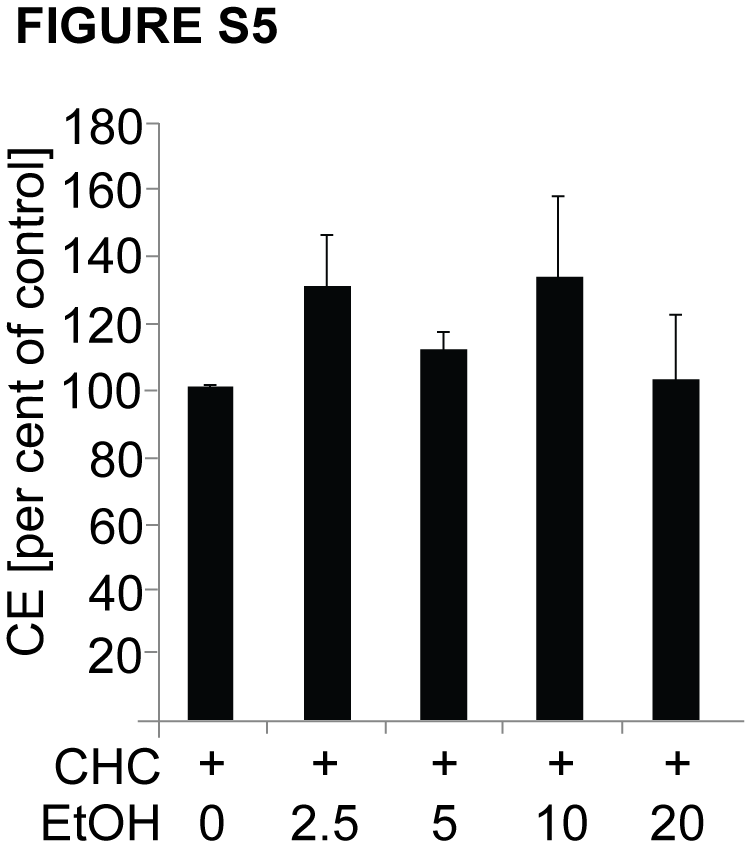

Supplement: Figure S5 — Ethanol has no effect on phagocytosis of cholesterol crystals by macrophages. Human primary macrophages were preincubated in the presence of the indicated concentrations of ethanol prior to addition of cholesterol crystals. Cellular cholesterol uptake was analyzed by thin layer chromatography by measuring cellular content of cholesteryl esters (CE). The data are expressed as per cent changes compared to macrophages incubated with cholesterol crystals in the absence of ethanol. The results are expressed as means ± s.e.m from 3 individual experiments. (TIF) [file pone.0078537.s005.tif]

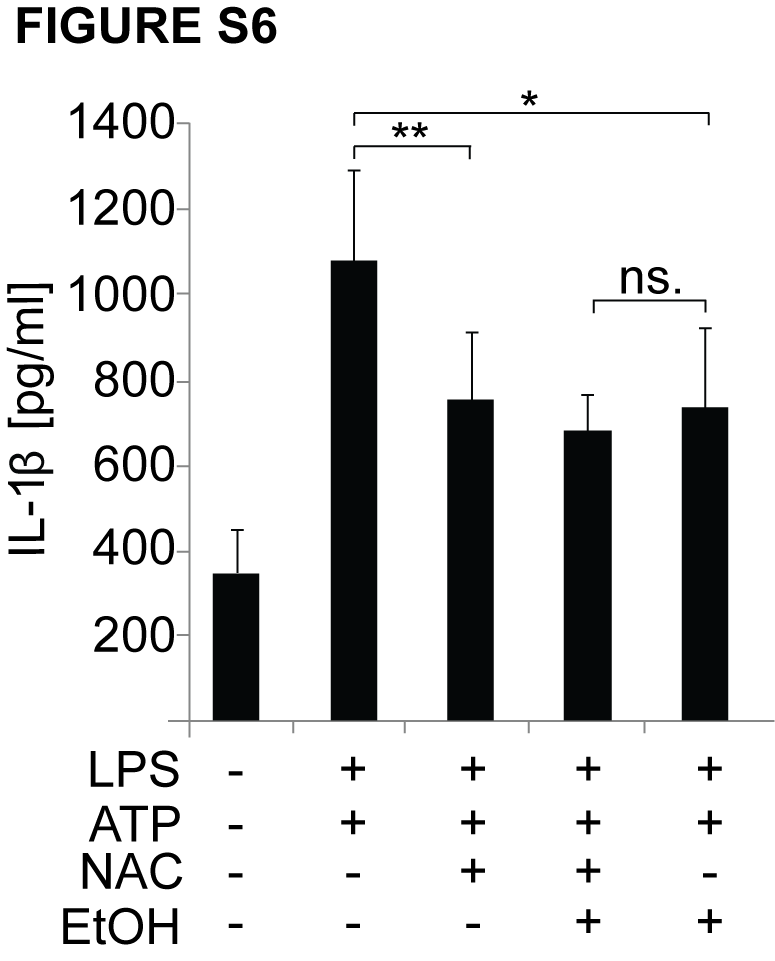

Supplement: Figure S6 — Scavenging reactive oxygen species do not influence the inhibitory effect of ethanol. LPS-primed THP-1 cells were first preincubated with or without the ROS scavenger N-acetyl-L-cysteine (NAC) then ethanol (final concentration 10‰ corresponding to 171 mM) was added, and finally the NLRP3 inflammasome was activated with ATP. Secretion of IL-1β into the culture medium was analyzed by ELISA. The results are expressed as means ± s.e.m. from 4 individual experiments, performed in duplicate. (TIF) [file pone.0078537.s006.tif]
