# Supplementary material for: Ethanol Inhibits Activation of NLRP3 and AIM2 Inflammasomes in Human Macrophages–A Novel Anti-Inflammatory Action of Alcohol
Source: PLoS One. 2013 Nov 11;8(11):e78537. doi: 10.1371/journal.pone.0078537 (PMC3823849; doi:10.1371/journal.pone.0078537)
Supplement: Table S1 — Primers and probes for quantitative real-time RT-PCR. (DOCX) [file pone.0078537.s007.docx]

Table S1

| Primers and probes for quantitative real-time RT-PCR | |
| --- | --- |
| IL1B-Forward | 5′-TTACAGTGGCAATGAGGATGAC-3′ |
| IL1B-Reverse | 5′-GTCGGAGATTCGTAGCTGGAT-3′ |
| IL1B fluorogenic probe | 5′-FAM-AACAGATGAAGTGCTCCTTCCAGGACC-BHQ1-3′ |
| NLRP3-Forward | 5′-GGAGAGACCTTTATGAGAAAGCAA-3′ |
| NLRP3-Reverse | 5′-GCTGTCTTCCTGGCATATCACA-3′ |
| NLRP3 fluorogenic probe | 5′-FAM-ACGTGCATTATCTGAACCCCACTTCGG-BHQ1-3′ |
| GAPDH-Forward | 5’-CCACATCGCTCAGACACCAT-3’ |
| GAPDH-Reverse | 5’-GGCAACAATATCCACTTTACCAGAG-3’ |
| GAPDH fluorogenic probe | 5’-FAM-CCAATACGACCAAATCCGTTGACTCC-BHQ1-3’ |
| AIM2-Forward | 5´-TGGCAAAACGTCTTCAGGAGG-3’ |
| AIM2-Reverse | 5´-AGCTTGACTTAGTGGCTTTGG-3’ |
| AIM2 fluorogenic probe | 5´-FAM-GTTGATAAGCAATACAAATCGG-BHQ1-3’ |
